# The avian gut microbiota: Diversity, influencing factors, and future directions

**DOI:** 10.3389/fmicb.2022.934272

**Published:** 2022-08-05

**Authors:** Fengfei Sun, Junfeng Chen, Kai Liu, Meizhen Tang, Yuewei Yang

**Affiliations:** School of Life Sciences, Qufu Normal University, Qufu, China

**Keywords:** captive birds, wild birds, influencing factors, gut microbiota, gut microbiome

## Abstract

The gut microbiota is viewed as the “second genome” of animals, sharing intricate relationships with their respective hosts. Because the gut microbial community and its diversity are affected by many intrinsic and extrinsic factors, studying intestinal microbes has become an important research topic. However, publications are dominated by studies on domestic or captive birds, while research on the composition and response mechanism of environmental changes in the gut microbiota of wild birds remains scarce. Therefore, it is important to understand the co-evolution of host and intestinal bacteria under natural conditions to elucidate the diversity, maintenance mechanisms, and functions of gut microbes in wild birds. Here, the existing knowledge of gut microbiota in captive and wild birds is summarized, along with previous studies on the composition and function, research methods employed, and factors influencing the avian gut microbial communities. Furthermore, research hotspots and directions were also discussed to identify the dynamics of the avian gut microbiota, aiming to contribute to studies of avian microbiology in the future.

## Introduction

The animal gut contains diverse intestinal microbes, achieving a dynamic balance with the host through mutual symbiosis and co-evolution during the evolutionary process (Costello et al., 2009; Mcfall-Ngai et al., 2013; Wang et al., 2019b). An increasing number of studies has shown that gut microbial communities can regulate several physiological functions, such as host digestion, absorption, metabolism, and immune function, as well as maintain host health in a variety of animals (Cantarel et al., 2012; Ezenwa et al., 2012; Cisek and Binek, 2014; Xu et al., 2020). Elucidating the underlying dynamics and regulatory mechanisms of animal intestinal microflora could further deepen our understanding of animal health and survival status, which has important guiding significance for animal protection and reproduction.

Birds are essential indicator organisms in several ecosystems with strong geographical dispersal ability and a wide distribution range. Strict environmental selection pressure, high energy consumption, contact with many intermediate organisms, and shortage of food resources during migration give birds unique intestinal microbial flora and complex immune functions (Wang et al., 2016a; Alexandra Garcia-Amado et al., 2018). Research on avian gut microbial communities has grown dramatically, owing to the increasing emphasis on bird populations and rapid developments in high-throughput sequencing (HTS) techniques (Waite and Taylor, 2015; Grond et al., 2018). The response and maintenance mechanisms of the avian gut microbiota to environmental changes have become a research hotspot for the sustainable survival and development of wild animal health.

**FIGURE 1:**
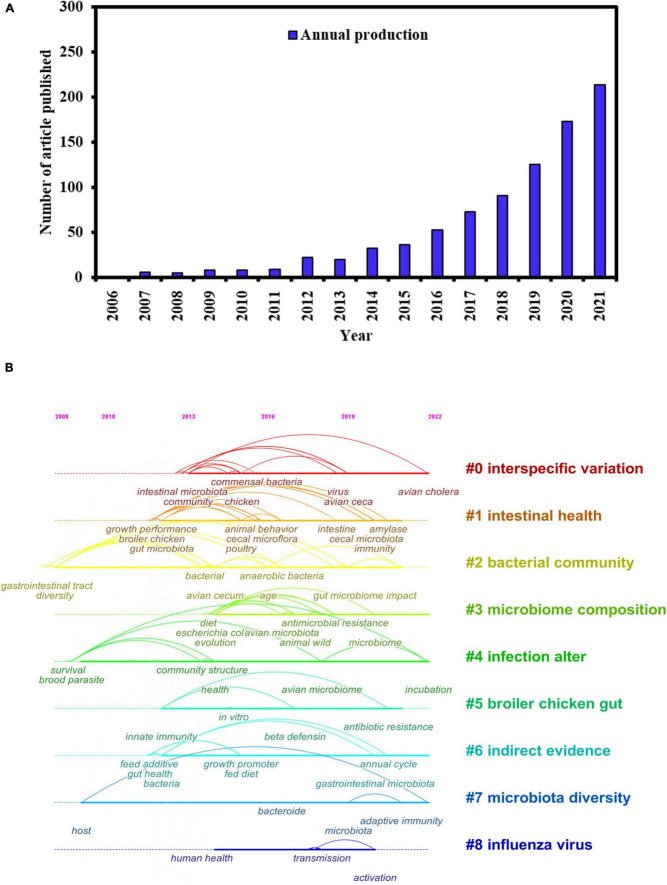
**(A)** Number of articles published on avian gut microbiota from 2006 to 2022. **(B)** The evolution of avian gut microbiota over the years.

Birds occupy a vital niche in the ecosystem, playing multiple ecological functions and possessing a complex gut microbial composition (Waite and Taylor, 2015). The annual number of publications of studies on avian gut microbes is shown in Figure 1A. In the past 16 years, research on the avian gut microbiota has markedly increased, especially in 2021, when more than 200 studies have been published. To gain a comprehensive understanding of previous research on bird gut microbes, a keyword clustering time map was created to visualize the research results on bird gut microbes over the past 16 years (Figure 1B). Interspecific variation, microbiome composition, infection alteration, and microbiota diversity have been the recent research hotspots, highlighting the direction for further in-depth research on these topics. Owing to the limitations of complex sampling methods and low DNA extraction yield under field conditions, the study of the microbiome in the avian gut is relatively under-investigated compared with many other vertebrates (Turnbaugh et al., 2007; Engel and Moran, 2013; Hildebrand et al., 2013). In this review, starting with the current research status on gut microbes in captive and wild birds, the research progress of gut microbiota in birds was systematically summarized in terms of composition and diversity, function, research methods used, and the factors influencing the intestinal microorganisms found in bird species. Research hotspots and future directions were also put forward to provide primary data and theoretical support for researchers in this field.

## Avian gut microbiota in captive and wild birds

**FIGURE 2:**
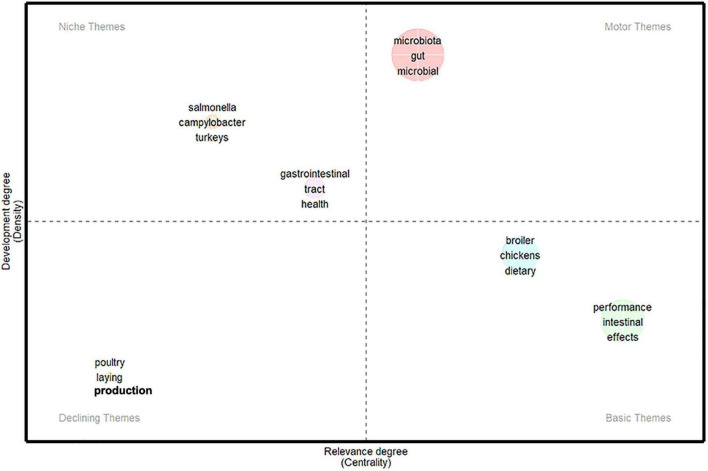
The thematic map of keywords analysis from 2006 to 2022.

As shown in Figure 2, avian gut microbial communities have received increasing attention over the past few decades. Accumulating evidence suggests that avian gut microbial communities can be shaped by the living conditions of their hosts. Species in captivity can have microbial community structures that differ significantly from wild species through environmental and dietary differences. As migratory birds have the habit of trans-regional migration, they can come into contact with different habitats and intermediate organisms. This contact makes the sources of their gut microbiota more diversified, providing more opportunities for the mutual transmission of gut microorganisms.

### Literature review of gut microbiota in captive birds

**FIGURE 3:**
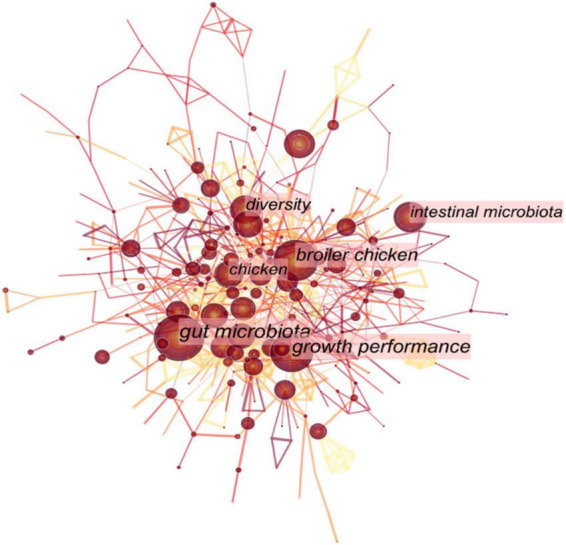
The keyword analysis in the topic of avian gut microbiota published manuscripts from 2006 to 2022.

Studies on the gut microbiome of captive birds have mainly focused on ornamental and economically significant birds, such as broilers (Gong et al., 2007), turkeys (Wilkinson et al., 2017), geese (Yang et al., 2018), ducks (Wang et al., 2018a), captive bar-headed geese (Wang et al., 2017), captive red-crowned cranes (Zhao et al., 2022), and pet birds (e.g., budgerigars and domestic canaries) (Garcia-Mazcorro et al., 2017; Figure 3 and Table 1). Food resources under captive conditions can have a significant impact on the avian gut microbiota. The simple gut microbiota composition of captive or domestic birds fed an immobilized diet has an insufficient stress response to external stimuli, resulting in a significant increase in the abundance of pathogenic groups in the bacterial community in their gut. Research on the microbial community of captive and domestic birds primarily focused on the effects of rearing conditions and viral infection on the microbial community (Wang et al., 2018a; Chen and Li, 2022), the immune function of gut microbiota in the defense against disease (Peng et al., 2021), and the influence of antibiotics and probiotics on avian gut intestinal microbes (Figueroa et al., 2020; Shi et al., 2020). In addition, the characteristics of the microbial composition in different regions of the gastrointestinal tract of poultry have also received significant attention (Gong et al., 2007; Wilkinson et al., 2017; Yang et al., 2018). A previous study investigated the gut microbial composition of Shaoxing ducks reared under different conditions and found distinct differences in their intestinal microflora composition (Wang et al., 2018a). Since the 20th century, frequent outbreaks of bird diseases (such as chicken colibacillosis and avian virus infection) have caused massive social, economic, and ecological problems (Massacci et al., 2018). For example, the avian leukosis virus was deemed harmful to poultry as it could change the gut microbial composition of Huiyang-bearing chickens and disrupt host–microbial homeostasis (Chen and Li, 2022). Additionally, the susceptibility of chickens to avian pathogenic *Escherichia coli* stimulation was increased with the depletion of gut microbiota, while acetate derived from the gut microbiota played a protective role during avian pathogenic *Escherichia coli* (APEC) stimulation (Peng et al., 2021). Supplementing ducks with *Lactobacillus* can mitigate gut microbial dysbiosis resulting from infection with duck *E. coli* 17 (Shi et al., 2020). Simultaneously, after treatment with avian influenza vaccines and anthelmintics in zoos, the gut microbiota of red-crowned cranes (*Grus japonensis*) might be disturbed in a short time but could recover to homeostasis over time (Zhao et al., 2022). The intestinal microflora can also have a protective function in the early life of chicks. The transfer of microbial communities between offspring and parents promotes the establishment of an early bacterial community balance and diversity in chicks and improves the stability of the microbial communities in their gut after H9N2 stress (Li et al., 2022).

**TABLE 1:** Influencing factors affecting avian gut microbiota in different living conditions.

Living condition	Affecting factors	Bird species	References
Captive	Avian leukosis virus subgroup J infection	Huiyang bearded chickens	Chen and Li, 2022
	Variation along the gastrointestinal tract	Chicken	Gong et al., 2007; Yan et al., 2019
		Turkeys	Wilkinson et al., 2017
		Goose	Yang et al., 2018
		Duck	Wang et al., 2018a
	Rearing conditions	Shaoxing ducks	Wang et al., 2018a
	Rearing conditions and age	Kakapo	Waite and Taylor, 2014
	Probiotics	Cherry Valley ducks	Shi et al., 2020
	Host	Chicken	Zhao et al., 2013
Wild	Age	Little penguin and short-tailed shearwater	Dewar et al., 2017
		Eurasian Kestrel	Zhou et al., 2020
		Chinstrap penguins	Barbosa et al., 2016
	Fasting	Little penguin and king penguins	Dewar et al., 2014b
	Sex and diet	Great Bustard	Liu et al., 2020; Gongora et al., 2021
	Diet	Great bustards and common cranes	Li et al., 2021
		Snow buntings, sanderlings, and pink-footed geese	Cho and Lee, 2020
	Nesting environment	Great tits	Teyssier et al., 2018
	Diet, island, and age	Kakapo	Perry et al., 2017
	Social interaction	Zebra finch	Chen et al., 2020
	Wintering areas	Black-necked crane	Wang et al., 2020
	Host	King penguin, gentoo penguin, macaroni penguin, and little penguin	Dewar et al., 2013
Captive vs Wild		Bar-headed goose	Wang et al., 2017
		Barn swallows	Turjeman et al., 2020
		Capercaillie	Wienemann et al., 2011
		Swan goose	Wang et al., 2016b

It is essential to protect biodiversity through the captive breeding of endangered wild birds. Research on the gut microbiota of threatened or endangered animals in captivity helps us understand their annual life cycle, which has important implications for disease prevention, control, and bird conservation. Surveying six species of birds raised at the Wildlife Conservation Center demonstrated that remarkable differences in avian gut microbial communities and dominant bacterial phyla exist, depending on the species (Gao et al., 2021). Simultaneously, the diet and living environment may also be important factors affecting the gut microbiota, and short-term changes in these factors have a relatively small influence on the avian gut microbial community. A higher abundance of Bacteroidetes was noted in the artificially reared Bar-headed goose (*Anser indicus*) compared to the wild group (Wang et al., 2017).

### Literature review of gut microbiota in wild birds

In recent years, research on the gut microbiota of wild birds has gained increasing attention. The gut microbiome of the South American hoatzin (*Opisthocomus hoazin*) (Godoy-Vitorino et al., 2012), the critically endangered kâkâpô (*Strigops habroptilus*) (Waite et al., 2014; Perry et al., 2017), the black vulture (*Coragyps atratus*) and turkey vulture (*Cathartes aura*) (Roggenbuck et al., 2014; Mendoza et al., 2018), passerine bird species (Hird et al., 2014; Bodawatta et al., 2018; Gadau et al., 2019; Chen et al., 2020), Anatidae birds (Wang et al., 2018b; Xiang et al., 2019), red-billed choughs (*Pyrrhocorax pyrrhocorax*) (Wang et al., 2019b), some penguins (Dewar et al., 2014b; Yew et al., 2018; Lee et al., 2019), and some water-bird species (Dewar et al., 2014a; Grond et al., 2014) have been reported (Table 1).

Wild birds have complex life-history characteristics, diverse dietary habits, unique mating systems, and long-distance migratory capacity, making their physiological activities face more substantial selective pressure, thereby contributing to the complexity of their gut microbiota. For example, the gut microbial development of short-tailed shearwater (*Ardenna tenuirostris*) and little penguin (*Eudyptula minor*) differed. The microbiota of short-tailed shearwater was relatively more stable throughout the development than that of the little penguin, which showed noticeable fluctuations (Dewar et al., 2017). Notably, the role of migratory behavior in the formation of gut microbiota in barn swallows (*Hirundo rustica*) has been investigated by sampling at the same location during autumn migration, indicating that there existed divergence in the intestinal microbe communities among migrant and resident subspecies, which may be attributed to the different breeding sites (Turjeman et al., 2020). Moreover, the effects of diet, age, and sex on the intestinal microbes in wild birds have also attracted extensive attention (Liu et al., 2020; Gongora et al., 2021; Li et al., 2021). Compared to populations that fed primarily on wild prey, the infection rates of *Salmonella* sp. were increased in the gut microbiota of red kites (*Milvus milvus*), which were fed a mixed diet consisting of wild prey and livestock carrion (Blanco, 2014). When comparing the gut microbial community of 8- and 15-day-old chicks of the great tits (*Parus major*), the gut microbial community diversity of the 15-day-old chicks was significantly lower than that of 8-day chicks, and the relative abundance of Firmicutes increased (Teyssier et al., 2018).

Migratory birds can become agents of infection for many diseases, transmitting antibiotic-resistant bacteria or as vectors of pathogens (Elmberg et al., 2017; Chung et al., 2018). In addition, flocking accelerates the spread of pathogenic microorganisms among sympathetic birds (Jourdain et al., 2007; Caron et al., 2010). Therefore, it is beneficial to understand the changes in the intestinal microflora upon direct or indirect contact between the host and other bird species. This information has important implications for the risk assessment of intestinal pathogenic microorganism interactions between wild birds and the conservation of endangered species.

## Microbial composition and functions

The avian gut core microbiota is generally very similar to that of other vertebrates at higher taxonomic levels. It appears to be generally predominated by Firmicutes and a small number of other bacterial phyla, such as Actinobacteria, Bacteroidetes, and Proteobacteria (Waite and Taylor, 2014; Grond et al., 2018; Wang et al., 2019b). This dominance is not surprising; it implies a relatively conservative group of microbial communities in the avian gut that allows better adaptation to natural conditions (Waite and Taylor, 2014). Contrary to the view that birds do not possess unique bacterial phyla, accumulating evidence has shown a noticeable difference in the gut microbes between birds and other vertebrates (Hird et al., 2014), especially at lower taxonomic levels (Godoy-Vitorino et al., 2008). The avian gut bacterial composition clustered apart from humans, insects, and fish and was closer to that of reptiles (Waite and Taylor, 2014). Furthermore, the diversity and composition of the gut microbiota among bird species can vary considerably. For example, the gut microbes of New Zealand kâkâpô consist of only a handful of phylotypes, whereas those of hoatzins belong to more than 40 phyla (Godoy-Vitorino et al., 2012). Therefore, it is necessary to compare the diversity in as many wild birds as possible for a more detailed description of the core microbiota in avian species.

Gut microbiota seems to be a crucial determinant of host health and physiology, as it serves as an “organ” that can provide a variety of essential functions for their hosts, such as vitamin synthesis, host metabolism, nutrient absorption, immune function, and organ development (Qin et al., 2010; Heijtza et al., 2011; Kohl, 2012; Al-Asmakh et al., 2014). Animal gut microflora can contribute to the decomposition of organic substances, such as carbohydrates and polysaccharides in the digestive tract, allowing them to absorb nutrients and participate in the metabolic processes in the host body (Bentley and Meganathan, 1982; Carroll et al., 2011). Microorganisms that provide enzymes have been isolated from chicken and turkey crops by degrading starch and monosaccharides that provide energy to the host (Bolton, 1965; Pinchasov and Noy, 1994; Pacheco et al., 2004). In addition, many uric acid-degrading microorganisms have been identified in the avian gut (Mead, 1989). Host nitrogen conservation has been shown to involve microorganism-induced uric acid metabolism, particularly in species that feed on a low-protein diet. Microorganisms specializing in uric acid metabolism have been detected in the guts of chickens, turkeys, common pheasants, and hermits (Barnes, 1972; Preest et al., 2003). Birds that feed on plants ingest toxic secondary metabolites, and the gut microbial communities that break down these toxicants are primarily concentrated in the crop and cecum. Microorganisms that can detoxify secondary plant metabolites have been proposed in the hoatzin crop (*Opisthocomus hoazin*) (Dearing et al., 2005). Furthermore, the gut microbiota of chickens can metabolize a variety of trichothecene mycotoxins (Young et al., 2007). Microbes colonizing the gastrointestinal tract are also essential for host immune system formation. The stable gut microflora is beneficial for improving host resistance, thereby decreasing the risk of infection by harmful foreign bacteria (Lee and Gemmell, 1972; Fuller, 1989; Umetsu et al., 2002).

## Research methods

### Sample collection

**FIGURE 4:**
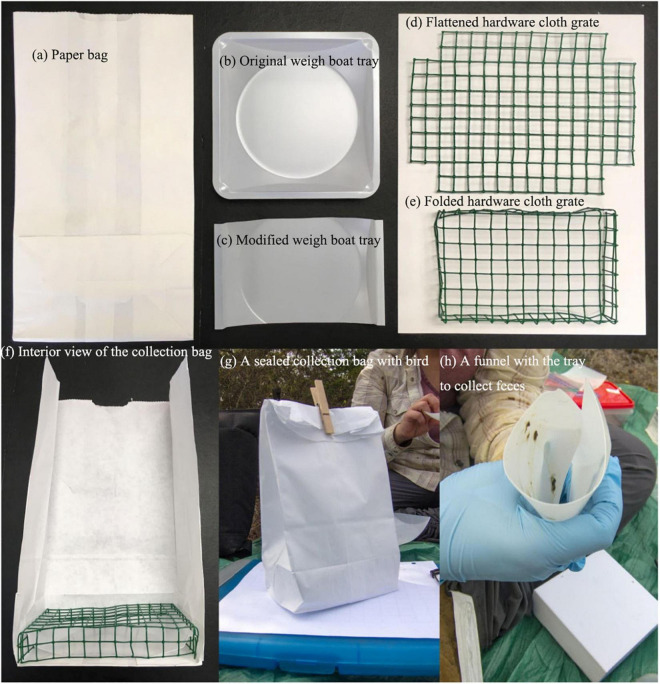
Schematic diagram for the non-invasive sample of feces using the collection kit [Reprinted from Knutie and Gotanda (2018)].

Several methods have been used to collect gut bacterial community samples from hosts, such as destructive sampling and non-invasive procedures. Destructive sampling involves euthanizing live birds prepared as museum samples, which may accurately reflect the composition of microorganisms in the different gut regions of these animals. However, this approach is not feasible when investigating the relationship of the gut microbiota to the fitness indicators or strategies based on the conservation of bird populations (Kohl et al., 2016). Currently, cloacal, fecal, and other non-lethal samples are the primary sources of samples for studying the gut microbiome in birds (Grond et al., 2018; Li et al., 2018; Youngblut et al., 2019). For example, sterile swabs were applied to obtain microbial samples from the cloaca of barn owls (*Tyto alba*) (Corl et al., 2020), and fresh feces samples were directly collected from the wintering area of the hooded crane (*Grus monacha*) and domestic geese (*Anser anser domesticus*) for the analysis of their gut microbial composition (Fu et al., 2020). Therefore, the method used to collect microbial samples depends on the species studied. This non-destructive method can be easily operated and has little impact on individual birds; it has also been widely applied in the research of intestinal microbes in wild birds. Multiple lines of evidence have demonstrated that the fecal microbial community is more reflective of the actual gut microbiota than cloacal samples (Videvall et al., 2018), making fecal samples a surrogate for studying the gastrointestinal microbiota (Yan et al., 2019). Recently, an innovative method for the non-invasive sampling of feces has attracted extensive attention (Figure 4; Knutie and Gotanda, 2018). With its advantages of low cost and ease of use, it is expected to be used in ecological sampling conditions. Notably, it can also be easily modified to suit various needs.

Sample preservation has always been a concern under field conditions. Storing bird feces or cloacal swab samples in liquid nitrogen or a −20°C refrigerator is widely used after sample collection to accurately reflect the composition of bird gut microbes (Corl et al., 2020). However, the need for liquid nitrogen and refrigerator storage increases the difficulty of working under field conditions. Recently, ethanol preservation methods have gradually gained attention (Bodawatta et al., 2020). Furthermore, a recent study has shown that the gut microbial composition of ethanol-preserved and frozen samples was highly similar to that of fresh samples and is also relatively stable over time (Hale et al., 2015).

### Traditional cultural methods

**FIGURE 5:**
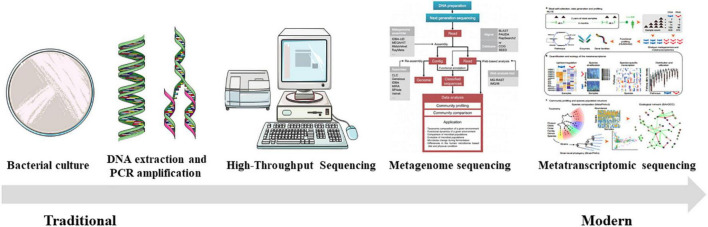
The evolution of commonly utilized techniques for studying the avian gut microbiome [the general strategy for the metagenomic approach was reprinted from Song et al. (2016) and the schematic diagram of metatranscriptomic sequencing was reprinted from Abu-Ali et al. (2018)].

Previous studies have used traditional culture methods that rely on media to perform phenotypic analysis of microorganisms by evaluating their morphological and biochemical characteristics (Henao-Mejia et al., 2012; Figure 5). Using conventional bacterial culture methods, the gut bacteria of the São Tomé thrush (*Turdus olivaceofuscus*), which is endemic on São Tomé Island in Africa, and the African thrush (*Turdus pelios*) found on the adjacent Gabon continent were cultured, indicating no appreciable differences in the diversity of culturable intestinal microflora between the two thrushes (Lobato et al., 2017). However, owing to the different growth characteristics of the host gut microbiota, the intestinal microbiota cultivated via traditional methods are mainly aerobic organisms that grow easily. At the same time, anaerobic bacteria are not evaluated, and the diversity of the intestinal microbial communities is underestimated. Therefore, there are limitations to studying dynamic changes in the gut microbiota (Pace, 1997; Stewart, 2012; Vocale et al., 2015).

### Traditional molecular biology techniques

With the development of bacterial genome research technologies in the late 20th century, molecular methods for identifying different microbial species based on 16S ribosomal RNA (16S rRNA) gene sequences have emerged (Wu et al., 2013). PCR-based amplification and identification of bacterial 16S rRNA maximized the classification and identification of bacteria, allowing a better understanding of the composition and diversity of bacteria. For example, a 16S rRNA gene library was constructed to evaluate the intestinal microbiota of turkeys (Lu and Domingo, 2008). In long-term evolutionary history, the hypervariable regions of bacterial 16S rRNA become mutated, resulting in significant differences in 16S rRNA gene sequences between bacteria. The difference in the length between these hypervariable fragments, or other specific nucleotide sequences, can be used for bacterial species identification (Muyzer, 1999; Inglis et al., 2012). However, there are limitations to using this method to discriminate between bacterial species (Muyzer, 1999). It is challenging to distinguish between bacteria with relatively small gene lengths and sequence differences. Simultaneously, it easily ignores bacteria with low abundance. Therefore, these methods have been eventually replaced with next-generation sequencing technologies.

### High-throughput sequencing

First-generation sequencing technology was invented by Frederick Sanger in the 1970s, which is also known as the dideoxy chain termination method (more popularly known as Sanger sequencing). The human genome has been sequenced using this technology, driving the development of the field of genomics (Sanger et al., 1977). The high cost and low sequencing volume of traditional Sanger sequencing technology limit its application in large-scale sequencing (Sanger et al., 1977). High-throughput sequencing (HTS) is a “next-generation sequencing technology” based on first-generation sequencing (Schuster, 2008). HTS can complete massive parallel sequencing runs, which has the advantages of low cost, high speed, and high throughput (Wheeler et al., 2008). As a powerful technique for evaluating intestinal microbiota, HTS can allow a more holistic study of the composition and function of gut microbes (Diaz-Sanchez et al., 2013). The advancement of HTS technologies, including the 454 pyrophosphate sequencing and Illumina sequencing platforms, has enabled a more efficient analysis of microbial communities (Swidsinski et al., 2002; Atarashi et al., 2011). High-throughput sequencing technology is widely used and has broad application prospects in studying microorganisms. Metagenome sequencing technology also has great potential for the functional prediction of avian gut microbes. It can analyze the community structure, gene function, and metabolic network of sample microbes through high-throughput sequencing of the whole-genome DNA (Garcovich et al., 2012). For instance, the composition, diversity, and function of the gut intestinal microflora in farmed and wild bar-headed geese have been analyzed using this technology (Wang et al., 2017). Furthermore, metatranscriptomic sequencing is commonly used to collect the transcripts of microorganisms in avian fecal samples and has received considerable attention. A recent study using metatranscriptomics demonstrated widespread antibiotic resistance in birds found in remote locations, such as Australia and Antarctica, and that exposure to human waste (even after sewage treatment) appears to affect avian wildlife access to ARGs (Marcelino et al., 2019). The rapid development of HTS techniques has brought revolutionary changes to microbial research and has extensively promoted the study of bird gut microbes.

## Influencing factors

**FIGURE 6:**
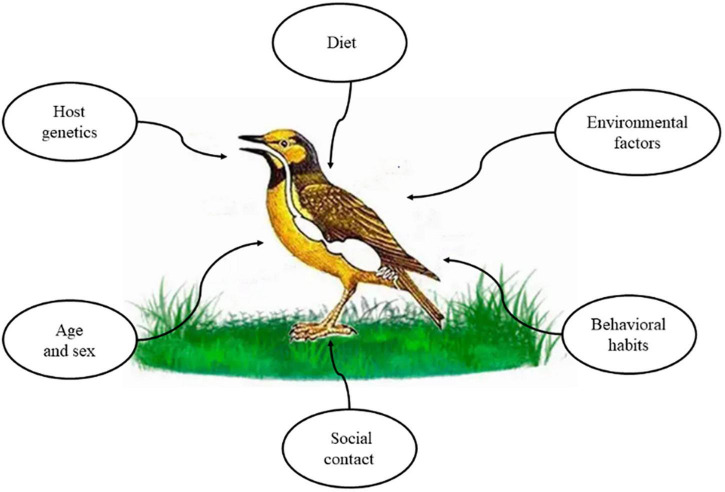
Main factors shaping avian intestinal microflora.

Birds, the largest branch of flying vertebrates, can be linked to geographically distant locations through migration and dispersal (Bauer and Hoye, 2014). Therefore, their gut microbial assemblage patterns may be influenced by a combination of intrinsic and extrinsic factors (Ruiz-Rodriguez et al., 2018). Intrinsic factors are closely related to the host, including host genetics, age, and sex, whereas extrinsic factors include dietary differences, behaviors, social relationships, and external environmental factors (Figure 6).

### Intrinsic factors

#### Host genetics

The genetic composition also significantly affects the microbial community of the animal gut, which the offspring can inherit through vertical transmission and co-evolution with the host (Baldo et al., 2017; Koskella et al., 2017). Therefore, from an evolutionary point of view, closely related species have high similarities in gut microbial community composition. Notably, genetically intact parental microbial communities are considered a critical reason for the phylogeny of microbial communities (Grond et al., 2014). In addition, differences in the species and evolutionary history also have a significant impact on animal gut bacterial communities (Ley et al., 2008). A study investigated the influence of 18 categorical variables concerning wild birds on their gut microbiota and pointed out that the host taxonomic category was the most crucial determinant, explaining the most significant variation in avian intestinal microbes (Hird et al., 2015). Similar results were observed in four penguin species (Dewar et al., 2013). The intestinal microflora of different species could vary significantly even when living in the same or similar environment (Lucas and Heeb, 2005). In the red knot (*Calidris canutus*) and ruddy turnstone (*Arenaria interpres*), only 10.87% of genera and 6.45% of OTUs in their gut bacterial communities were shared even if they lived in similar habitat in Delaware Bay, United States (Grond et al., 2014). The habitats of these water birds were similar, but the gut microbial community structure showed species specificity. Simultaneously, nestlings of magpies (*Pica pica*) and spotted cuckoos (*Clamator glandarius*) with shared environmental conditions during growth showed evident differences in their cloacal microbial assemblages, suggesting that phylogenetic components act as determining factors in the formation of the intestinal microflora (Ruiz-Rodriguez et al., 2018). Furthermore, host taxonomy has been shown to be the most important determinant of the gut microbial community in birds (Waite and Taylor, 2014). Overall, these studies suggest that host genetics may outweigh environmental influences in determining gut microbial colonization (Fraune and Bosch, 2007).

#### Age and sex

The age of the host also influences the gut microbiota, contributing to significant differences among individuals (Bibbo et al., 2016; Org et al., 2016). Microbial colonization in the gut of birds occurs after egg hatching, and the colonization process may occur through diverse pathways (Kohl, 2012; Wang et al., 2019a). The composition of the intestinal microbiota differs significantly between chicks and early adults (Gonzalez-Braojos et al., 2012; Van Dongen et al., 2013). The bacterial community in adulthood is more stable and shows higher diversity than chicks in the early stages of life. This may be due to the effects of reproductive hormones, which could be implicated in the formation of bacterial communities (Jiménez et al., 2008; Hamady and Knight, 2009; Leclaire et al., 2014). The gastrointestinal microbial community of chinstrap penguins (*Pygoscelis antarctica*) differed considerably between chicks and adults. Firmicutes had a higher relative abundance in chicks than in adults. In contrast, Bacteroidetes and Proteobacteria were comparatively more abundant in adults; hence, the bacterial community in adults is more diverse and affluent than that in chicks (Barbosa et al., 2016).

Sex differences have a determining effect on the intestinal microflora of wild birds; however, there are only a few studies that documented the relationship between sex and gut microbiota in wild birds, which require relatively more data for detection (Kreisinger et al., 2015; Capunitan et al., 2020). This may be due to the difficulty of sampling both sexes in the wild (Liu et al., 2020). Compared to males, female Great Bustards (which feed on rice and peanuts) were colonized more by Firmicutes and less by Bacteroidetes, and their microbial diversity and evenness were higher (Liu et al., 2020). The influence of sex on the gut microbiota has also been illustrated in brown-headed cowbirds (*Molothrus ater*) (Hird et al., 2014) and broiler chickens (Lumpkins et al., 2008; Lee et al., 2017). Significant changes in gut microbes occurred after the animals were paired, and these changes were sex-biased. After pairing, male fecal samples contained more intestinal microbiota than females. Intriguingly, females of these species could transmit more microflora to their social partners than males (Zhu et al., 2020).

### Extrinsic factors

#### Diet

Ingestion of different types of food may also be a pathway for the colonization of host gut microbiota (Grond et al., 2017). Dietary differences can lead to extensive changes in host gut microbial richness and diversity, as significant differences in the gut microbiota have been found between carnivores, omnivores, and herbivores (Ley et al., 2008). As a striking example, there is a low gut microbiome diversity in vultures, which could be linked to their carrion-based diet (Roggenbuck et al., 2014). The gut microbial community of three Arctic species of snow buntings (*Plectrophenax nivalis*) residing in the same habitat was previously compared. Their gut microbial community was similar to sanderlings (*Calidris alba*) and quite different from that of pink-footed geese (*Anser brachyrhynchus*). This was probably because snow bunting and sanderlings shared the same diet. The intestinal microbial structure of pink-footed geese, which was associated with their herbivorous diet, was quite distinct from those of the other two birds (Cho and Lee, 2020). Furthermore, the diversity of gut microbial communities was significantly reduced when birds were fed urban food compared with individuals that were fed rural food (Teyssier et al., 2020). Dietary differences are supposed to play an integral role in shaping the gut microbial environment; hence, permanent changes in the diet may induce the colonization of new gut microbes, increasing the diversity and abundance of beneficial microflora (Gubert et al., 2020).

#### Environmental factors

Environmental spatial heterogeneity has a strong impact on avian microbial communities, which plays a dominant role in shaping gut microflora, sometimes overweighing genetic factors (Hird et al., 2014). The geographic location of the host and its accompanying changes in climate, flora, fauna, and other habitats affect the microbial pool in the local environment, thereby exerting a considerable influence on the host gut microbes (Kropáèková et al., 2017; Song et al., 2020). Swainson’s thrushes (*Catharus ustulatus*) and gray catbirds (*Dumetella carolinensis*) are some of the most striking examples of how environmental factors affect host gut microbiota, particularly compared with host genetics. There were distinct differences in the gut microbiome between these two species during spring migration and before crossing during fall (Lewis et al., 2016). A current study investigated the gut microbes of eight shorebird species living in the Arctic and sub-Arctic regions of North America. It highlighted that the natural conditions of the local environment played an indispensable role in shaping the intestinal microbial community in wild birds compared with phylogenetic factors (Grond et al., 2019). There were also significant differences in the goose gut microbiota between species grown in breeding and wintering areas affected by human activities. Compared to breeding areas with low levels of human activity, swan samples from overwintering areas have differences in species abundance and interaction network topology, and the enrichment of pathogenic bacteria is significantly different.

It has previously been confirmed that habitat disturbance and exposure to humans can significantly influence the microbes found in avian intestines (Phillips et al., 2018). For example, compared with a breeding area with low levels of human activity, the gut microbiota found in swan geese (*Anser cygnoides*) sampled from wintering areas differed in terms of species abundance and interaction network topologies and showed a distinctive enrichment of pathogenic bacteria (Wu et al., 2018). Additionally, a previous study pointed out that urbanization significantly altered the composition of intestinal microflora in house sparrows (*Passer domesticus*). More microorganisms from the phylum Proteobacteria, which are relevant in all types of mammalian intestinal and extra-intestinal diseases, were present in the gut of urban house sparrows (Gadau et al., 2019). Overall, the impact of human activities on host habitats can disrupt host-gut microbial community relationships.

#### Behavioral habits

Most birds can fly and migrate long distances, searching for the most suitable habitat. Compared with resident birds, migratory birds have a wider range of activities and can come into contact with different environments; therefore, they could obtain more microorganisms from their environment. However, previous studies have shown that the microbial diversity in the feathers of migratory birds was lower than that of resident birds, emphasizing the critical influence of a single environment on microbial diversity (Bisson et al., 2009). This may be because resident birds are exposed to various microbial communities during foraging behavior on the ground, whereas migratory birds use a single habitat and space (Merkel et al., 2006; Ley et al., 2008; Johnson et al., 2010). Furthermore, the persistence of gut microbes in migratory birds is currently poorly studied. The changes in the gut microbiota present across different times, and geographic locations may not be reflected in the different migratory stages of these birds. Owing to the constantly changing dietary resources in migratory birds, they may ingest various microorganisms associated with other food sources. During migration, the host gut microbiota of migratory birds become relatively similar within and between species, indicating that food resources or environments in different regions are essential factors in changing the host gut microbiota (Lewis et al., 2017).

#### Social contact

Social contact can mediate the acquisition and exchange of microflora between individuals of similar and different species, affecting the composition of their gut microbial community (Moller et al., 2009; Levin et al., 2016). A wide variety of bird social behaviors promote the mutual transmission of gut microbes among different individuals, spreading pathogenic microorganisms and providing a benefit by transferring symbionts (Lombardo, 2008; Ezenwa and Williams, 2014; Grond et al., 2014; Ryu et al., 2014). Additionally, to our knowledge, parental care has a considerable influence on the gut microbial community of their offspring, which can be achieved by altering the bacterial pathogen loads in their eggs. Notably, parental saliva also plays an essential role during feeding, transmitting microbiota to chicks (Kyle and Kyle, 2004; Brandl et al., 2014). For example, the gut microbes of zebra finch (*Taeniopygia guttata*) chicks are primarily obtained from nests coated with parental microbes (Chen et al., 2020). It has been reported that sexual contact can also alter the animal gut microbial community. For example, in red-winged blackbirds (*Agelaius phoeniceus*) and black-legged kittiwakes (*Rissa tridactyla*), kittiwakes artificially deterred from fertilization resulted in increasingly different cloacal microbiota in mating partners, with females showing decreased cloacal diversity and reduced numbers of bacteria shared with males (Westneat and Birch Rambo, 2000; White et al., 2010). In common lizards, polygamous females had a higher gut microbiota richness than monogamous females. They also had a more diverse microbiota composition, suggesting that sexual behavior can transmit a greater diversity of bacteria (White et al., 2011).

In conclusion, the establishment and maintenance of the avian gut microflora result from complicated interactions between host-specific and external factors. Based on the studies summarized in this review, host-specific factors, such as age, sex, and genetics, significantly influence the gut microbial composition of different bird species. However, the extent to which the host regulates the intestinal microbial community and function and its underlying mechanisms require further investigation. During evolution, investigating the continuous trajectories of intestinal microbial communities in birds with different life histories has practical implications in determining whether and when birds acquire stable microbial communities. Furthermore, maternal and environmental factors can be separated by manipulating the prenatal environment or by reciprocal transplantation experiments to distinguish the effects of genetic and environmental factors on the intestinal microflora community. Dietary differences in birds are associated with local seasonal food resources, and relevant studies should be performed to evaluate whether seasonal changes in intestinal microbes in wild birds depend on changes in their diet. Additionally, sex segregation outside the breeding season in many birds exposes them to different living conditions. The impact of differential migration on microbial exposure between males and females, which can be performed by sampling during non-breeding seasons, remains unclear. Regarding extrinsic factors, it is necessary to understand how the gut microbiota promotes host adaptation in birds. This requires monitoring the intestinal microbiota of birds in the field and exploring the response mechanism of their intestinal microbiota to the external environment. More research is needed to evaluate the interaction between host-specific and environmental factors in determining the community, diversity, function, and importance of intestinal microbes.

## Conclusion and future prospects

With recent advancements in HTS techniques, more publications are driven by the increased understanding of the crucial determinants of intestinal microbes in wild birds. Accumulating evidence implies that research on avian gut microbes lags behind that on other vertebrates, and notably, this gap is rapidly closing. As we have discussed, an increasing number of wild birds are receiving considerable attention from researchers, and more importantly, it is expected that research on the bird microbiome will soon expand to include more bird species. Birds are ideal subjects for studying the co-evolution and adaptive evolution of animal gut microbiota complexes. As a group of vertebrates with strong adaptability to radiation, they have strong migratory abilities, extremely diverse ecological niches, and are sensitive to environmental changes, representing an evolutionarily successful lineage. Based on the evidence presented regarding avian gut microbiota, several prospects are proposed, as follows:

First, a substantial body of evidence shows that avian gut microbes contain core phyla similar to mammalian intestinal microbes. However, many of the actual functions of avian intestinal microbes are closely related to evolutionary specialization. The development of metagenome sequencing techniques and metatranscriptomic sequencing technology has provided unprecedented insights into the community-level functions of the gut microbiota and interactions between the environment and the avian gut.

Second, migratory birds played a pivotal role in spreading the avian influenza virus, causing substantial economic losses and posing a threat to human safety. The transmission of microorganisms among wild birds and poultry has become an urgent concern that deserves attention. Strikingly, research on pathogenic species of intestinal microflora is of great significance to the conservation of birds.

Third, environmental threats, such as habitat degradation and loss, interspecific competition and human disturbance, and harsh climatic conditions, cause birds and their corresponding gut microbiota to face immense survival pressure. Therefore, it is also essential to investigate the spatiotemporal changes in the gut microbes of wild birds and their impact on their respective hosts. Furthermore, more studies should investigate how birds adjust their gut microbes to adapt to changing living conditions.

Given the current knowledge summarized in this review, research on avian gut microbiota is essential for understanding the survival status of wild bird populations, providing crucial information in protecting them, especially endangered species. It also provides an essential theoretical basis for epidemiological research on pathogens.

## Author contributions

FS: conceptualization, methodology, data curation, and writing – original draft. JC: conceptualization and data curation – review and editing. KL: data curation. MT: methodology and writing – review and editing. YY: conceptualization, data curation, and writing – review and editing. All authors contributed to the article and approved the submitted version.

## References

[B1] Abu-Ali G. S. Mehta R. S. Lloyd-Price J. Mallick H. Branck T. Ivey K. L. (2018). Metatranscriptome of human faecal microbial communities in a cohort of adult men. *Nat. Microbiol.* 3 356–366. 10.1038/s41564-017-0084-4 29335555 PMC6557121

[B2] Al-Asmakh M. Stukenborg J.-B. Reda A. Anuar F. Strand M.-L. Hedin L. (2014). The Gut Microbiota and Developmental Programming of the Testis in Mice. *PLoS One* 9:e103809. 10.1371/journal.pone.0103809 25118984 PMC4132106

[B3] Alexandra Garcia-Amado M. Shin H. Sanz V. Lentino M. Margarita Martinez L. Contreras M. (2018). Comparison of gizzard and intestinal microbiota of wild neotropical birds. *PLoS One* 13:e0194857. 10.1371/journal.pone.0194857 29579092 PMC5868825

[B4] Atarashi K. Tanoue T. Shima T. Imaoka A. Kuwahara T. Momose Y. (2011). Induction of Colonic Regulatory T Cells by Indigenous Clostridium Species. *Science* 331 337–341. 10.1126/science.1198469 21205640 PMC3969237

[B5] Baldo L. Lluis Pretus J. Lluis Riera J. Musilova Z. Nyom A. R. B. Salzburger W. (2017). Convergence of gut microbiotas in the adaptive radiations of African cichlid fishes. *ISME J.* 11 1975–1987. 10.1038/ismej.2017.62 28509910 PMC5560477

[B6] Barbosa A. Balague V. Valera F. Martinez A. Benzal J. Motas M. (2016). Age-Related Differences in the Gastrointestinal Microbiota of Chinstrap Penguins (Pygoscelis antarctica). *PLoS One* 11:e0153215. 10.1371/journal.pone.0153215 27055030 PMC4824521

[B7] Barnes E. M. (1972). The avian intestinal flora with particular reference to the possible ecological significance of the cecal anaerobic bacteria. *Am. J. Clin. Nutr.* 25 1475–1479. 10.1093/ajcn/25.12.1475 4346128

[B8] Bauer S. Hoye B. J. (2014). Migratory Animals Couple Biodiversity and Ecosystem Functioning Worldwide. *Science* 344:1242552. 10.1126/science.1242552 24700862

[B9] Bentley R. Meganathan R. (1982). Biosynthesis of vitamin K (menaquinone) in bacteria. *Microbiol. Rev.* 46 241–280. 10.1128/mr.46.3.241-280.1982 6127606 PMC281544

[B10] Bibbo S. Ianiro G. Giorgio V. Scaldaferri F. Masucci L. Gasbarrini A. (2016). The role of diet on gut microbiota composition. *Eur. Rev. Med. Pharmacol. Sci.* 20 4742–4749. 27906427

[B11] Bisson I.-A. Marra P. P. Burtt E. H. Jr. Sikaroodi M. Gillevet P. M. (2009). Variation in Plumage Microbiota Depends on Season and Migration. *Microb. Ecol.* 58 212–220. 10.1007/s00248-009-9490-3 19212698

[B12] Blanco G. (2014). Influence of diet on the gastrointestinal flora of wintering red kites. *Eur. J. Wildl. Res.* 60 695–698. 10.1007/s10344-014-0820-5

[B13] Bodawatta K. H. Puzejova K. Sam K. Poulsen M. Jonsson K. A. (2020). Cloacal swabs and alcohol bird specimens are good proxies for compositional analyses of gut microbial communities of Great tits (Parus major). *Animal Microbiome* 2 9–9. 10.1186/s42523-020-00026-8 33499943 PMC7807456

[B14] Bodawatta K. H. Sam K. Jonsson K. A. Poulsen M. (2018). Comparative Analyses of the Digestive Tract Microbiota of New Guinean Passerine Birds. *Front. Microbiol.* 9:1830. 10.3389/fmicb.2018.01830 30147680 PMC6097311

[B15] Bolton W. (1965). Digestion in the crop of the fowl. *Br. Poult. Sci.* 6 97–102. 10.1080/00071666508415561 5834520

[B16] Brandl H. B. Van Dongen W. F. D. Darolova A. Kristofik J. Majtan J. Hoi H. (2014). Composition of Bacterial Assemblages in Different Components of Reed Warbler Nests and a Possible Role of Egg Incubation in Pathogen Regulation. *PLoS One* 9:e114861. 10.1371/journal.pone.0114861 25493434 PMC4262450

[B17] Cantarel B. L. Lombard V. Henrissat B. (2012). Complex Carbohydrate Utilization by the Healthy Human Microbiome. *PLoS One* 7:e28742. 10.1371/journal.pone.0028742 22719820 PMC3374616

[B18] Capunitan D. C. Johnson O. Terrill R. S. Hird S. M. (2020). Evolutionary signal in the gut microbiomes of 74 bird species from Equatorial Guinea. *Mol. Ecol.* 29 829–847. 10.1111/mec.15354 31943484

[B19] Caron A. De Garine-Wichatitsky M. Gaidet N. Chiweshe N. Cumming G. S. (2010). Estimating Dynamic Risk Factors for Pathogen Transmission Using Community-Level Bird Census Data at the Wildlife/Domestic Interface. *Ecol. Soc.* 15 299–305. 10.5751/ES-03547-150325 30174746

[B20] Carroll I. M. Ringel-Kulka T. Keku T. O. Chang Y.-H. Packey C. D. Sartor R. B. (2011). Molecular analysis of the luminal- and mucosal-associated intestinal microbiota in diarrhea-predominant irritable bowel syndrome. *Am. J. Physiol. Gastrointest. Liver Physiol.* 301 799–807. 10.1152/ajpgi.00154.2011 21737778 PMC3220325

[B21] Chen C.-Y. Chen C.-K. Chen Y.-Y. Fang A. Shaw G. T.-W. Hung C.-M. (2020). Maternal gut microbes shape the early-life assembly of gut microbiota in passerine chicks via nests. *Microbiome* 8:129. 10.1186/s40168-020-00896-9 32917256 PMC7488855

[B22] Chen Y. Li H. (2022). Avian leukosis virus subgroup J infection influences the gut microbiota composition in Huiyang bearded chickens. *Lett. Appl. Microbiol.* 74 344–353. 10.1111/lam.13617 34825389

[B23] Cho H. Lee W. Y. (2020). Interspecific comparison of the fecal microbiota structure in three Arctic migratory bird species. *Ecol. Evol.* 10 5582–5594. 10.1002/ece3.6299 32607176 PMC7319242

[B24] Chung D. M. Ferree E. Simon D. M. Yeh P. J. (2018). Patterns of bird–bacteria associations. *Ecohealth* 15 627–641. 10.1007/s10393-018-1342-5 29948415 PMC6521974

[B25] Cisek A. A. Binek M. (2014). Chicken intestinal microbiota function with a special emphasis on the role of probiotic bacteria. *Pol. J. Vet. Sci.* 17 385–394. 10.2478/pjvs-2014-0057 24988871

[B26] Corl A. Charter M. Rozman G. Toledo S. Turjeman S. Kamath P. L. (2020). Movement ecology and sex are linked to barn owl microbial community composition. *Mol. Ecol.* 29 1358–1371. 10.1111/mec.15398 32115796

[B27] Costello E. K. Lauber C. L. Hamady M. Fierer N. Gordon J. I. Knight R. (2009). Bacterial Community Variation in Human Body Habitats Across Space and Time. *Science* 326 1694–1697. 10.1126/science.1177486 19892944 PMC3602444

[B28] Dearing M. D. Foley W. J. Mclean S. (2005). The influence of plant secondary metabolites on the nutritional ecology of herbivorous terrestrial vertebrates. *Annu. Rev. Ecol. Evol. Syst.* 36 169–189. 10.1146/annurev.ecolsys.36.102003.152617

[B29] Dewar M. L. Arnould J. P. Allnutt T. R. Crowley T. Krause L. Reynolds J. (2017). Microbiota of little penguins and short-tailed shearwaters during development. *PLoS One* 12:e0183117. 10.1371/journal.pone.0183117 28806408 PMC5555571

[B30] Dewar M. L. Arnould J. P. Y. Dann P. Trathan P. Groscolas R. Smith S. (2013). Interspecific variations in the gastrointestinal microbiota in penguins. *Microbiologyopen* 2 195–204. 10.1002/mbo3.66 23349094 PMC3584224

[B31] Dewar M. L. Arnould J. P. Y. Krause L. Trathan P. Dann P. Smith S. C. (2014b). Influence of Fasting during Moult on the Faecal Microbiota of Penguins. *PLoS One* 9:e99996. 10.1371/journal.pone.0099996 24979619 PMC4076183

[B32] Dewar M. L. Arnould J. P. Y. Krause L. Dann P. Smith S. C. (2014a). Interspecific variations in the faecal microbiota of Procellariiform seabirds. *FEMS Microbiol. Ecol.* 89 47–55. 10.1111/1574-6941.12332 24684257

[B33] Diaz-Sanchez S. Hanning I. Pendleton S. D’souza D. (2013). Next-generation sequencing: the future of molecular genetics in poultry production and food safety. *Poult. Sci.* 92 562–572. 10.3382/ps.2012-02741 23300324

[B34] Elmberg J. Berg C. Lerner H. Waldenström J. Hessel R. (2017). Potential disease transmission from wild geese and swans to livestock, poultry and humans: a review of the scientific literature from a One Health perspective. *Infect. Ecol. Epidemiol.* 7:1300450. 10.1080/20008686.2017.1300450 28567210 PMC5443079

[B35] Engel P. Moran N. A. (2013). The gut microbiota of insects - diversity in structure and function. *Fems Microbiol. Rev.* 37 699–735. 10.1111/1574-6976.12025 23692388

[B36] Ezenwa V. O. Gerardo N. M. Inouye D. W. Medina M. Xavier J. B. (2012). Animal Behavior and the Microbiome. *Science* 338 198–199. 10.1126/science.1227412 23066064

[B37] Ezenwa V. O. Williams A. E. (2014). Microbes and animal olfactory communication: where do we go from here? *BioEssays* 36 847–854. 10.1002/bies.201400016 24986361

[B38] Figueroa T. Bessiere P. Coggon A. Bouwman K. M. Van Der Woude R. Delverdier M. (2020). The Microbiota Contributes to the Control of Highly Pathogenic H5N9 Influenza Virus Replication in Ducks. *J. Virol.* 94 e289–e220. 10.1128/JVI.00289-20 32102887 PMC7199410

[B39] Fraune S. Bosch T. C. G. (2007). Long-term maintenance of species-specific bacterial microbiota in the basal metazoan Hydra. *Proc. Natl. Acad. Sci. U.S.A.* 104 13146–13151. 10.1073/pnas.0703375104 17664430 PMC1934924

[B40] Fu R. Xiang X. Dong Y. Cheng L. Zhou L. (2020). Comparing the intestinal bacterial communies of sympatric wintering Hooded Crane (*Grus monacha*) and Domestic Goose (*Anser anser domesticus*). *Avian Res.* 11:13. 10.1186/s40657-020-00195-9

[B41] Fuller R. (1989). Probiotics in man and animals. *J. Appl. Bacteriol.* 66 365–378. 10.1111/j.1365-2672.1989.tb05105.x 2666378

[B42] Gadau A. Meli’sa S. C. Mayek R. Giraudeau M. Mcgraw K. J. Whisner C. M. (2019). A comparison of the nutritional physiology and gut microbiome of urban and rural house sparrows (*Passer domesticus*). *Comp. Biochem. Physiol. B Biochem. Mol. Biol.* 237:110332. 10.1016/j.cbpb.2019.110332 31461685

[B43] Gao L. Liu L. Du C. Hou Q. (2021). Comparative Analysis of Fecal Bacterial Microbiota of Six Bird Species. *Front. Vet. Sci.* 8:791287. 10.3389/fvets.2021.791287 34957285 PMC8692710

[B44] Garcia-Mazcorro J. F. Castillo-Carranza S. A. Guard B. Gomez-Vazquez J. P. Dowd S. E. Brigthsmith D. J. (2017). Comprehensive Molecular Characterization of Bacterial Communities in Feces of Pet Birds Using 16S Marker Sequencing. *Microb. Ecol.* 73 224–235. 10.1007/s00248-016-0840-7 27568186

[B45] Garcovich M. Zocco M. A. Roccarina D. Ponziani F. R. Gasbarrini A. (2012). Prevention and treatment of hepatic encephalopathy: focusing on gut microbiota. *World J. Gastroentero.* 18 5–12. 10.3748/wjg.v18.i46.6693 23239905 PMC3520156

[B46] Godoy-Vitorino F. Goldfarb K. C. Karaoz U. Leal S. Garcia-Amado M. A. Hugenholtz P. (2012). Comparative analyses of foregut and hindgut bacterial communities in hoatzins and cows. *ISME J.* 6 531–541. 10.1038/ismej.2011.131 21938024 PMC3280141

[B47] Godoy-Vitorino F. Ley R. E. Gao Z. Pei Z. Ortiz-Zuazaga H. Pericchi L. R. (2008). Bacterial community in the crop of the hoatzin, a neotropical folivorous flying bird. *Appl. Environ. Microbiol.* 74 5905–5912. 10.1128/AEM.00574-08 18689523 PMC2565963

[B48] Gong J. Si W. Forster R. J. Huang R. Yu H. Yin Y. (2007). 16S rRNA gene-based analysis of mucosa-associated bacterial community and phylogeny in the chicken gastrointestinal tracts: from crops to ceca. *FEMS Microbiol. Ecol.* 59 147–157. 10.1111/j.1574-6941.2006.00193.x 17233749

[B49] Gongora E. Elliott K. H. Whyte L. (2021). Gut microbiome is affected by inter-sexual and inter-seasonal variation in diet for thick-billed murres (Uria lomvia). *Sci. Rep.* 11:1200. 10.1038/s41598-020-80557-x 33441848 PMC7806582

[B50] Gonzalez-Braojos S. Vela A. I. Ruiz-De-Castaneda R. Briones V. Moreno J. (2012). Age-related changes in abundance of enterococci and *Enterobacteriaceae* in Pied Flycatcher (*Ficedula hypoleuca*) nestlings and their association with growth. *J. Ornithol.* 153 181–188. 10.1007/s10336-011-0725-y

[B51] Grond K. Lanctot R. B. Jumpponen A. Sandercock B. K. (2017). Recruitment and establishment of the gut microbiome in arctic shorebirds. *Fems Microbiol. Ecol.* 93:fix142. 10.1093/femsec/fix142 29069418

[B52] Grond K. Ryu H. Baker A. J. Domingo J. W. S. Buehler D. M. (2014). Gastro-intestinal microbiota of two migratory shorebird species during spring migration staging in Delaware Bay USA. *J. Ornithol.* 155 969–977. 10.1007/s10336-014-1083-3

[B53] Grond K. Sandercock B. K. Jumpponen A. Zeglin L. H. (2018). The avian gut microbiota: community, physiology and function in wild birds. *J. Avian Biol.* 49:e01788. 10.1111/jav.01788

[B54] Grond K. Santo Domingo J. W. Lanctot R. B. Jumpponen A. Bentzen R. L. Boldenow M. L. (2019). Composition and Drivers of Gut Microbial Communities in Arctic-Breeding Shorebirds. *Front. Microbiol.* 10:2258. 10.3389/fmicb.2019.02258 31649627 PMC6795060

[B55] Gubert C. Kong G. Renoir T. Hannan A. J. (2020). Exercise, diet and stress as modulators of gut microbiota: implications for neurodegenerative diseases. *Neurobiol. Dis.* 134:104621. 10.1016/j.nbd.2019.104621 31628992

[B56] Hale V. L. Tan C. L. Knight R. Amato K. R. (2015). Effect of preservation method on spider monkey (Ateles geoffroyi) fecal microbiota over 8 weeks. *J. Microbiol. Methods* 113 16–26. 10.1016/j.mimet.2015.03.021 25819008

[B57] Hamady M. Knight R. (2009). Microbial community profiling for human microbiome projects: tools, techniques, and challenges. *Genome Res.* 19 1141–1152. 10.1101/gr.085464.108 19383763 PMC3776646

[B58] Heijtza R. D. Wang S. Anuar F. Qian Y. Bjorkholm B. Samuelsson A. (2011). Normal gut microbiota modulates brain development and behavior. *Proc. Natl. Acad. Sci. U.S.A.* 108 3047–3052. 10.1073/pnas.1010529108 21282636 PMC3041077

[B59] Henao-Mejia J. Elinav E. Jin C. Hao L. Mehal W. Z. Strowig T. (2012). Inflammasome-mediated dysbiosis regulates progression of NAFLD and obesity. *Nature* 482 179–185. 10.1038/nature10809 22297845 PMC3276682

[B60] Hildebrand F. Anh N. Brinkman B. Yunta R. G. Cauwe B. Vandenabeele P. (2013). Inflammation-associated enterotypes, host genotype, cage and inter-individual effects drive gut microbiota variation in common laboratory mice. *Genome Biol.* 14:R4. 10.1186/gb-2013-14-1-r4 23347395 PMC4053703

[B61] Hird S. M. Carstens B. C. Cardiff S. Dittmann D. L. Brumfield R. T. (2014). Sampling locality is more detectable than taxonomy or ecology in the gut microbiota of the brood-parasitic Brown-headed Cowbird (Molothrus ater). *PeerJ* 2:e321. 10.7717/peerj.321 24711971 PMC3970801

[B62] Hird S. M. Sanchez C. Carstens B. C. Brumfield R. T. (2015). Comparative Gut Microbiota of 59 Neotropical Bird Species. *Front. Microbiol.* 6:1403. 10.3389/fmicb.2015.01403 26733954 PMC4685052

[B63] Inglis G. D. Thomas M. C. Thomas D. K. Kalmokoff M. L. Brooks S. P. J. Selinger L. B. (2012). Molecular Methods to Measure Intestinal Bacteria: a Review. *J. AOAC Int.* 95 5–23. 10.5740/jaoacint.SGE_Inglis 22468337

[B64] Jiménez E. Marín M. L. Martín R. Odriozola J. M. Olivares M. Xaus J. (2008). Is meconium from healthy newborns actually sterile? *Res. Microbiol.* 159 187–193. 10.1016/j.resmic.2007.12.007 18281199

[B65] Johnson M. Clarkson P. Goldstein M. I. Haig S. M. Lanctot R. B. Tessler D. F. (2010). Seasonal Movements Winter range use, and migratory connectivity of the black oystercatcher. *Condor* 112 731–743. 10.1525/cond.2010.090215

[B66] Jourdain E. Gauthier-Clerc M. Bicout D. Sabatier P. (2007). Bird migration routes and risk for pathogen dispersion into western Mediterranean wetlands. *Emerg. Infect. Dis.* 13 365–372. 10.3201/eid1303.060301 17552088 PMC2725901

[B67] Knutie S. A. Gotanda K. M. (2018). A Non-invasive Method to Collect Fecal Samples from Wild Birds for Microbiome Studies. *Microb. Ecol.* 76 851–855. 10.1007/s00248-018-1182-4 29623358

[B68] Kohl K. D. (2012). Diversity and function of the avian gut microbiota. *J. Comp. Physiol. B* 182 591–602. 10.1007/s00360-012-0645-z 22246239

[B69] Kohl K. D. Connelly J. W. Dearing M. D. Forbey J. S. (2016). Microbial detoxification in the gut of a specialist avian herbivore, the Greater Sage-Grouse. *Fems Microbiol. Lett.* 363:fnw144. 10.1093/femsle/fnw144 27242374

[B70] Koskella B. Hall L. J. Metcalf C. J. E. (2017). The microbiome beyond the horizon of ecological and evolutionary theory. *Nat. Ecol. Evol.* 1 1606–1615. 10.1038/s41559-017-0340-2 29038487

[B71] Kreisinger J. Cizkova D. Kropackova L. Albrecht T. (2015). Cloacal Microbiome Structure in a Long-Distance Migratory Bird Assessed Using Deep 16sRNA Pyrosequencing. *PLoS One* 10:e0137401. 10.1371/journal.pone.0137401 26360776 PMC4567286

[B72] Kropáèková L. Tìšickı M. Albrecht T. Kubovèiak J. Èížková D. Tomášek O. (2017). Codiversification of gastrointestinal microbiota and phylogeny in passerines is not explained by ecological divergence. *Mol. Ecol.* 26 5292–5304. 10.1111/mec.14144 28401612

[B73] Kyle G. Z. Kyle P. D. (2004). *Rehabilitation and Conservation of Chimney Swifts (Chaetura pelagica).* Austin: Driftwood Wildlife Association

[B74] Leclaire S. Nielsen J. F. Drea C. M. (2014). Bacterial communities in meerkat anal scent secretions vary with host sex, age, and group membership. *Behav. Ecol.* 25 996–1004. 10.1093/beheco/aru074

[B75] Lee A. Gemmell E. (1972). Changes in the mouse intestinal microflora during weaning: role of volatile fatty acids. *Infect. Immun.* 5 1–7. 10.1128/iai.5.1.1-7.1972 4656353 PMC422310

[B76] Lee K.-C. Kil D. Y. Sul W. J. (2017). Cecal microbiome divergence of broiler chickens by sex and body weight. *J. Microbiol.* 55 939–945. 10.1007/s12275-017-7202-0 29214491

[B77] Lee W. Y. Cho H. Kim M. Tripathi B. M. Jung J.-W. Chung H. (2019). Faecal microbiota changes associated with the moult fast in chinstrap and gentoo penguins. *PLoS One* 14:e0216565. 10.1371/journal.pone.0216565 31067284 PMC6505947

[B78] Levin I. I. Zonana D. M. Fosdick B. K. Song S. J. Knight R. Safran R. J. (2016). Stress response, gut microbial diversity and sexual signals correlate with social interactions. *Biol. Lett.* 12:20160352. 10.1098/rsbl.2016.0352 27354713 PMC4938059

[B79] Lewis W. B. Moore F. R. Wang S. (2016). Characterization of the gut microbiota of migratory passerines during stopover along the northern coast of the Gulf of Mexico. *J. Avian Biol.* 47 659–668. 10.1111/jav.00954

[B80] Lewis W. B. Moore F. R. Wang S. (2017). Changes in gut microbiota of migratory passerines during stopover after crossing an ecological barrier. *AUK* 134 137–145. 10.1642/AUK-16-120.1 7633414

[B81] Ley R. E. Hamady M. Lozupone C. Turnbaugh P. J. Ramey R. R. Bircher J. S. (2008). Evolution of mammals and their gut microbes. *Science* 320 1647–1651. 10.1126/science.1155725 18497261 PMC2649005

[B82] Li C. Liu Y. Gong M. Zheng C. Zhang C. Li H. (2021). Diet-induced microbiome shifts of sympatric overwintering birds. *Appl. Microbiol. Biotechnol.* 105 5993–6005. 10.1007/s00253-021-11448-y 34272578

[B83] Li X. Bi R. Xiao K. Roy A. Zhang Z. Chen X. (2022). Hen raising helps chicks establish gut microbiota in their early life and improve microbiota stability after H9N2 challenge. *Microbiome* 10:14. 10.1186/s40168-021-01200-z 35074015 PMC8785444

[B84] Li Y. Yang H. Xu L. Wang Z. Zhao Y. Chen X. (2018). Effects of dietary fiber levels on cecal microbiota composition in geese. *Asian-australas. J. Anim. Sci.* 31 1285–1290. 10.5713/ajas.17.0915 29381893 PMC6043456

[B85] Liu G. Meng D. Gong M. Li H. Wen W. Wang Y. (2020). Effects of Sex and Diet on Gut Microbiota of Farmland-Dependent Wintering Birds. *Front. Microbiol.* 11:587873. 10.3389/fmicb.2020.587873 33262746 PMC7688461

[B86] Lobato E. Geraldes M. Melo M. Doutrelant C. Covas R. (2017). Diversity and composition of cultivable gut bacteria in an endemic island bird and its mainland sister species. *Symbiosis* 71 155–164. 10.1007/s13199-016-0419-6

[B87] Lombardo M. P. (2008). Access to mutualistic endosymbiotic microbes: an underappreciated benefit of group living. *Behav. Ecol. Sociobiol.* 62 479–497. 10.1007/s00265-007-0428-9

[B88] Lu J. Domingo J. S. (2008). Turkey fecal microbial community structure and functional gene diversity revealed by 16S rRNA gene and metagenomic sequences. *J. Microbiol.* 46 469–477. 10.1007/s12275-008-0117-z 18974945

[B89] Lucas F. S. Heeb P. (2005). Environmental factors shape cloacal bacterial assemblages in great tit Parus major and blue tit P. caeruleus nestlings. *J. Avian Biol.* 36 510–516. 10.1111/j.0908-8857.2005.03479.x

[B90] Lumpkins B. S. Batal A. B. Lee M. (2008). The effect of gender on the bacterial community in the gastrointestinal tract of broilers. *Poult. Sci.* 87 964–967. 10.3382/ps.2007-00287 18420988

[B91] Marcelino V. R. Wille M. Hurt A. C. González-Acuña D. Klaassen M. Schlub T. E. (2019). Meta-transcriptomics reveals a diverse antibiotic resistance gene pool in avian microbiomes. *BMC Biol.* 17:31. 10.1186/s12915-019-0649-1 30961590 PMC6454771

[B92] Massacci F. R. Magistrali C. F. Cucco L. Curcio L. Bano L. Mangili P. (2018). Characterization of of Pasteurella multocida involved in rabbit infections. *Vet. Microbiol.* 213 66–72. 10.1016/j.vetmic.2017.11.023 29292006

[B93] Mcfall-Ngai M. Hadfield M. G. Bosch T. C. G. Carey H. V. Domazet-Loso T. Douglas A. E. (2013). Animals in a bacterial world, a new imperative for the life sciences. *Proc. Natl. Acad. Sci. U.S.A.* 110 3229–3236. 10.1073/pnas.1218525110 23391737 PMC3587249

[B94] Mead G. C. (1989). Microbes of the avian cecum: types present and substrates utilized. *J. Exp. Zool.* 3 48–54. 10.1002/jez.1402520508 2575127

[B95] Mendoza M. L. Z. Roggenbuck M. Manzano Vargas K. Hansen L. H. Brunak S. Gilbert M. T. P. (2018). Protective role of the vulture facial skin and gut microbiomes aid adaptation to scavenging. *Acta Vet. Scand.* 60:61. 10.1186/s13028-018-0415-3 30309375 PMC6182802

[B96] Merkel F. R. Mosbech A. Sonne C. Flagstad A. Falk K. Jamieson S. E. (2006). Local movements, home ranges and body condition of Common Eiders Somateria mollissima wintering in Southwest Greenland. *Ardea* 94 639–650.

[B97] Moller A. P. Czirjak G. A. Heeb P. (2009). Feather micro-organisms and uropygial antimicrobial defences in a colonial passerine bird. *Funct. Ecol.* 23 1097–1102. 10.1111/j.1365-2435.2009.01594.x

[B98] Muyzer G. (1999). DGGE/TGGE a method for identifying genes from natural ecosystems. *Curr. Opin. Microbiol.* 2 317–322. 10.1016/S1369-5274(99)80055-1 10383868

[B99] Org E. Mehrabian M. Parks B. W. Shipkova P. Liu X. Drake T. A. (2016). Sex differences and hormonal effects on gut microbiota composition in mice. *Gut Microbes* 7 313–322. 10.1080/19490976.2016.1203502 27355107 PMC4988450

[B100] Pace N. R. (1997). A molecular view of microbial diversity and the biosphere. *Science* 276 734–740. 10.1126/science.276.5313.734 9115194

[B101] Pacheco M. A. GarcÍa-Amado M. A. Bosque C. DomÍnguez-Bello M. G. (2004). Bacteria in the crop of the seed-eating green-rumped parrotlet. *Condor* 106 139–143. 10.1093/condor/106.1.139

[B102] Peng L.-Y. Shi H.-T. Gong Z.-X. Yi P.-F. Tang B. Shen H.-Q. (2021). Protective effects of gut microbiota and gut microbiota-derived acetate on chicken colibacillosis induced by avian pathogenic escherichia coli. *Vet. Microbiol.* 261:109187. 10.1016/j.vetmic.2021.109187 34399296

[B103] Perry E. K. Digby A. Taylor M. W. (2017). The Low-Diversity Fecal Microbiota of the Critically Endangered Kakapo Is Robust to Anthropogenic Dietary and Geographic Influences. *Front. Microbiol.* 8:2033. 10.3389/fmicb.2017.02033 29104565 PMC5655120

[B104] Phillips J. N. Berlow M. Derryberry E. P. (2018). The Effects of Landscape Urbanization on the Gut Microbiome: an Exploration Into the Gut of Urban and Rural White-Crowned Sparrows. *Front. Ecol. Evol.* 6:148. 10.3389/fevo.2018.00148

[B105] Pinchasov Y. Noy Y. (1994). Early postnatal amylolysis in the gastrointestinal tract of turkey poults *Meleagris gallopavo*. *Comp. Biochem. Physiol.* 107 221–226. 10.1016/0300-9629(94)90297-6

[B106] Preest M. R. Folk D. G. Beuchat C. A. (2003). Decomposition of nitrogenous compounds by intestinal bacteria in hummingbirds. *Auk* 120 1091–1101. 10.1642/0004-8038(2003)120[1091:DONCBI]2.0.CO;2

[B107] Qin J. Li R. Raes J. Arumugam M. Burgdorf K. S. Manichanh C. (2010). A human gut microbial gene catalogue established by metagenomic sequencing. *Nature* 464 59–65. 10.1038/nature08821 20203603 PMC3779803

[B108] Roggenbuck M. Schnell I. B. Blom N. Baelum J. Bertelsen M. F. Ponten T. S. (2014). The microbiome of New World vultures. *Nat. Commun.* 5:5498. 10.1038/ncomms6498 25423494

[B109] Ruiz-Rodriguez M. Martin-Vivaldi M. Martinez-Bueno M. Jose Soler J. (2018). Gut Microbiota of Great Spotted Cuckoo Nestlings is a Mixture of Those of Their Foster Magpie Siblings and of Cuckoo Adults. *Genes* 9:381. 10.3390/genes9080381 30060541 PMC6115760

[B110] Ryu H. Grond K. Verheijen B. Elk M. Buehler D. M. Domingo J. W. S. (2014). Intestinal Microbiota and Species Diversity of *Campylobacter* and *Helicobacter* spp. in Migrating Shorebirds in Delaware Bay. *Appl. Environ. Microbiol.* 80 1838–1847. 10.1128/AEM.03793-13 24413599 PMC3957654

[B111] Sanger F. Nicklen S. Coulson A. R. (1977). DNA sequencing with chain-terminating inhibitors. *Proc. Natl. Acad. Sci. U.S.A.* 74 5463–5467. 10.1073/pnas.74.12.5463 271968 PMC431765

[B112] Schuster S. C. (2008). Next-generation sequencing transforms today’s biology. *Nat. Methods* 5 16–18. 10.1038/nmeth1156 18165802

[B113] Shi S. Qi Z. Jiang W. Quan S. Sheng T. Tu J. (2020). Effects of probiotics on cecal microbiome profile altered by duck *Escherichia coli* 17 infection in Cherry Valley ducks. *Microb. Pathog.* 138:103849. 10.1016/j.micpath.2019.103849 31704465

[B114] Song H. J. Lee J. Graf L. Rho M. Qiu H. Bhattacharya D. (2016). A novice’s guide to analyzing NGS-derived organelle and metagenome data. *Algae* 31 137–154. 10.4490/algae.2016.31.6.5

[B115] Song M. Chan A. T. Sun J. (2020). Influence of the Gut Microbiome Diet, and Environment on Risk of Colorectal Cancer. *Gastroenterology* 158 322–340. 10.1053/j.gastro.2019.06.048 31586566 PMC6957737

[B116] Stewart E. J. (2012). Growing Unculturable Bacteria. *J. Bacteriol.* 194 4151–4160. 10.1128/JB.00345-12 22661685 PMC3416243

[B117] Swidsinski A. Ladhoff A. Pernthaler A. Swidsinski S. Loening–Baucke V. Ortner M. (2002). Mucosal flora in inflammatory bowel disease. *Gastroenterology* 122 44–54. 10.1053/gast.2002.30294 11781279

[B118] Teyssier A. Lens L. Matthysen E. White J. (2018). Dynamics of Gut Microbiota Diversity During the Early Development of an Avian Host: evidence From a Cross-Foster Experiment. *Front. Microbiol.* 9:1524. 10.3389/fmicb.2018.01524 30038608 PMC6046450

[B119] Teyssier A. Matthysen E. Hudin N. S. De Neve L. White J. Lens L. (2020). Diet contributes to urban-induced alterations in gut microbiota: experimental evidence from a wild passerine. *Proc. Royal Soc. B Biol. Sci.* 287:20192182. 10.1098/rspb.2019.2182 32019440 PMC7031670

[B120] Turjeman S. Corl A. Wolfenden A. Tsalyuk M. Lublin A. Choi O. (2020). Migration, pathogens and the avian microbiome: a comparative study in sympatric migrants and residents. *Mol. Ecol.* 29 4706–4720. 10.1111/mec.15660 33001530

[B121] Turnbaugh P. J. Ley R. E. Hamady M. Fraser-Liggett C. M. Knight R. Gordon J. I. (2007). The human microbiome project. *Nature* 449 804–810. 10.1038/nature06244 17943116 PMC3709439

[B122] Umetsu D. T. Mcintire J. J. Akbari O. Macaubas C. Dekruyff R. H. (2002). Asthma: an epidemic of dysregulated immunity. *Nat. Immunol.* 3 715–720. 10.1038/ni0802-715 12145657

[B123] Van Dongen W. F. D. White J. Brandl H. B. Moodley Y. Merkling T. Leclaire S. (2013). Age-related differences in the cloacal microbiota of a wild bird species. *BMC Ecol.* 13:11. 10.1186/1472-6785-13-11 23531085 PMC3668179

[B124] Videvall E. Strandh M. Engelbrecht A. Cloete S. Cornwallis C. K. (2018). Measuring the gut microbiome in birds: comparison of faecal and cloacal sampling. *Mol. Ecol. Res.* 18 424–434. 10.1111/1755-0998.12744 29205893

[B125] Vocale C. Rimoldi S. G. Pagani C. Grande R. Pedna F. Arghittu M. (2015). Comparative evaluation of the new xTAG GPP multiplex assay in the laboratory diagnosis of acute gastroenteritis. Clinical assessment and potential application from a multicentre Italian study. *Int. J. Infect. Dis.* 34 33–37. 10.1016/j.ijid.2015.02.011 25749649

[B126] Waite D. W. Eason D. K. Taylor M. W. (2014). Influence of Hand Rearing and Bird Age on the Fecal Microbiota of the Critically Endangered Kakapo. *Appl. Environ. Microbiol.* 80 4650–4658. 10.1128/AEM.00975-14 24837385 PMC4148800

[B127] Waite D. W. Taylor M. W. (2014). Characterizing the avian gut microbiota: membership, driving influences, and potential function. *Front. Microbiol.* 5:223–233. 10.3389/fmicb.2014.00223 24904538 PMC4032936

[B128] Waite D. W. Taylor M. W. (2015). Exploring the avian gut microbiota: current trends and future directions. *Front. Microbiol.* 6:673. 10.3389/fmicb.2015.00673 26191057 PMC4490257

[B129] Wang S. Chen L. He M. Shen J. Li G. Tao Z. (2018a). Different rearing conditions alter gut microbiota composition and host physiology in Shaoxing ducks. *Sci. Rep.* 8:7387. 10.1038/s41598-018-25760-7 29743727 PMC5943461

[B130] Wang W. Liu Y. Yang Y. Wang A. Sharshov K. Li Y. (2018b). Comparative analyses of the gut microbiota among three different wild geese species in the genus Anser. *J. Basic Microbiol.* 58 543–553. 10.1002/jobm.201800060 29668076

[B131] Wang W. Cao J. Li J.-R. Yang F. Li Z. Li L.-X. (2016a). Comparative analysis of the gastrointestinal microbial communities of bar-headed goose (Anser indicus) in different breeding patterns by high-throughput sequencing. *Microbiol. Res.* 182 59–67. 10.1016/j.micres.2015.10.003 26686614

[B132] Wang W. Zheng S. Sharshov K. Cao J. Sun H. Yang F. (2016b). Distinctive gut microbial community structure in both the wild and farmed Swan goose (Anser cygnoides). *J. Basic Microbiol.* 56 1299–1307. 10.1002/jobm.201600155 27365218

[B133] Wang W. Wang A. Yang Y. Wang F. Liu Y. Zhang Y. (2019b). Composition, diversity and function of gastrointestinal microbiota in wild red-billed choughs (Pyrrhocorax pyrrhocorax). *Int. Microbiol.* 22 491–500. 10.1007/s10123-019-00076-2 31020476

[B134] Wang W. Sharshov K. Zhang Y. Gui L. (2019a). Age-related Changes in the Cloacal Microbiota of Bar-headed Geese (Anser indicus). *Kafkas Universitesi Veteriner Fakultesi Dergisi* 25 575–582.

[B135] Wang W. Wang F. Li L. Wang A. Sharshov K. Druzyaka A. (2020). Characterization of the gut microbiome of black-necked cranes (Grus nigricollis) in six wintering areas in China. *Arch. Microbiol.* 202 983–993. 10.1007/s00203-019-01802-0 31901964

[B136] Wang W. Zheng S. Sharshov K. Sun H. Yang F. Wang X. (2017). Metagenomic profiling of gut microbial communities in both wild and artificially reared Bar-headed goose (Anser indicus). *MicrobiologyOpen* 6:e00429. 10.1002/mbo3.429 27998035 PMC5387313

[B137] Westneat D. F. Birch Rambo T. (2000). Copulation exposes female Red-winged Blackbirds to bacteria in male semen. *J. Avian Biol.* 31 1–7. 10.1034/j.1600-048X.2000.310101.x 11841302

[B138] Wheeler D. A. Srinivasan M. Egholm M. Shen Y. Chen L. Mcguire A. (2008). The complete genome of an individual by massively parallel DNA sequencing. *Nature* 452 872–876. 10.1038/nature06884 18421352

[B139] White J. Mirleau P. Danchin E. Mulard H. Hatch S. A. Heeb P. (2010). Sexually transmitted bacteria affect female cloacal assemblages in a wild bird. *Ecol. Lett.* 13 1515–1524. 10.1111/j.1461-0248.2010.01542.x 20961376 PMC3772342

[B140] White J. Richard M. Massot M. Meylan S. (2011). Cloacal Bacterial Diversity Increases with Multiple Mates: evidence of Sexual Transmission in Female Common Lizards. *PLoS One* 6:e22339. 10.1371/journal.pone.0022339 21811590 PMC3141023

[B141] Wienemann T. Schmitt-Wagner D. Meuser K. Segelbacher G. Schink B. Brune A. (2011). The bacterial microbiota in the ceca of Capercaillie (Tetrao urogallus) differs between wild and captive birds. *Syst. Appl. Microbiol.* 34 542–551. 10.1016/j.syapm.2011.06.003 21889862

[B142] Wilkinson T. J. Cowan A. A. Vallin H. E. Onime L. A. Oyama L. B. Cameron S. J. (2017). Characterization of the Microbiome along the Gastrointestinal Tract of Growing Turkeys. *Front. Microbiol.* 8:1089. 10.3389/fmicb.2017.01089 28690591 PMC5479886

[B143] Wu N. Yang X. Zhang R. Li J. Xiao X. Hu Y. (2013). Dysbiosis Signature of Fecal Microbiota in Colorectal Cancer Patients. *Microb. Ecol.* 66 462–470. 10.1007/s00248-013-0245-9 23733170

[B144] Wu Y. Yang Y. Cao L. Yin H. Xu M. Wang Z. (2018). Habitat environments impacted the gut microbiome of long-distance migratory swan geese but central species conserved. *Sci. Rep.* 8:13314. 10.1038/s41598-018-31731-9 30190564 PMC6127342

[B145] Xiang X. Zhang F. Fu R. Yan S. Zhou L. (2019). Significant differences in bacterial and potentially pathogenic communities between sympatric hooded crane and greater white-fronted goose. *Front. Microbiol.* 10:163. 10.3389/fmicb.2019.00163 30804919 PMC6370644

[B146] Xu H. Cai Z. Wang Y. Wu D. Rong W. Zhang G. (2020). Effects and pathophysiological significance of intestinal flora on the enteric neuro-endocrine-immune system. *Sheng Li Xue Bao* 72 347–360. 32572432

[B147] Yan W. Sun C. Zheng J. Wen C. Ji C. Zhang D. (2019). Efficacy of Fecal Sampling as a Gut Proxy in the Study of Chicken Gut Microbiota. *Front. Microbiol.* 10:2126. 10.3389/fmicb.2019.02126 31572332 PMC6753641

[B148] Yang H. Xiao Y. Gui G. Li J. Wang J. Li D. (2018). Microbial community and short-chain fatty acid profile in gastrointestinal tract of goose. *Poult. Sci.* 97 1420–1428. 10.3382/ps/pex438 29365165

[B149] Yew W. C. Pearce D. A. Dunn M. J. Adlard S. Alias S. A. Samah A. A. (2018). Links between bacteria derived from penguin guts and deposited guano and the surrounding soil microbiota. *Polar Biol.* 41 269–281. 10.1007/s00300-017-2189-x

[B150] Young J. C. Zhou T. Yu H. Zhu H. Gong J. (2007). Degradation of trichothecene mycotoxins by chicken intestinal microbes. *Food Chem. Toxicol.* 45 136–143. 10.1016/j.fct.2006.07.028 17011105

[B151] Youngblut N. D. Reischer G. H. Walters W. Schuster N. Walzer C. Stalder G. (2019). Host diet and evolutionary history explain different aspects of gut microbiome diversity among vertebrate clades. *Nat. Commun.* 10:2200. 10.1038/s41467-019-10191-3 31097702 PMC6522487

[B152] Zhao L. Wang G. Siegel P. He C. Wang H. Zhao W. (2013). Quantitative Genetic Background of the Host Influences Gut Microbiomes in Chickens. *Sci. Rep.* 3:1163. 10.1038/srep01163 23362462 PMC3557447

[B153] Zhao X. Ye W. Xu W. Xu N. Zheng J. Chen R. (2022). Changes in the Diversity and Composition of Gut Microbiota of Red-Crowned Cranes (*Grus japonensis*) after Avian Influenza Vaccine and Anthelmintic Treatment. *Animals* 12:1183. 10.3390/ani12091183 35565609 PMC9099658

[B154] Zhou L. Huo X. Liu B. Wu H. Feng J. (2020). Comparative Analysis of the Gut Microbial Communities of the Eurasian Kestrel (Falco tinnunculus) at Different Developmental Stages. *Front. Microbiol.* 11:592539. 10.3389/fmicb.2020.592539 33391209 PMC7775371

[B155] Zhu L. Clayton J. B. Van Haute M. J. S. Yang Q. Hassenstab H. R. Mustoe A. C. (2020). Sex Bias in Gut Microbiome Transmission in Newly Paired Marmosets (Callithrix jacchus). *mSystems* 5 e00910–19. 10.1128/mSystems.00910-19 32209720 PMC7093826

